# 921. Acute HAV Infection in an Inpatient Psychiatry Unit

**DOI:** 10.1093/ofid/ofab466.1116

**Published:** 2021-12-04

**Authors:** Gregory Weston, Carmel Boland-Reardon, Renee Rhoden, Rose Ogbonna, Surksha Sirichand, Inessa Gendlina, Inessa Gendlina, Marilou Corpuz

**Affiliations:** 1 Montefiore Medical Center and Albert Einstein College of Medicine, Bronx, New York; 2 Montefiore Medical Center, Bronx, NY; 3 Albert Einstein College of Medicine, Bronx, NY

## Abstract

**Background:**

The incidence of hepatitis A virus (HAV) infection has been rising in the US since 2016, and in New York State since 2019. New York City has also seen an increase of HAV infection among high risk populations. We present a case of acute HAV infection in an inpatient psychiatry unit which has its own unique isolation and management challenges.

**Methods:**

A patient was admitted on 3/21/21 from a group home. He developed abdominal pain, diarrhea and vomiting on 4/15, with elevated liver function tests (LFT). He was transferred to Medicine on 4/17 and HAV IgM and IgG resulted positive on 4/18. Visitation to the unit has been halted for over a year, and no outside food has been allowed. The patient has not been observed to have any sexual exposure to others.

**Investigation:**

Exposure window: 15 days prior to start of symptoms. Patients in the unit were screened for symptoms, tested for HAV IgM/IgG, LFTs. Discharged patients were contacted and referred straight for vaccination (difficult to have multiple visits). Staff members with contact to the unit were screened, via email and phone calls. If no previous vaccination and there was presence of exposure or symptoms, staff were referred to Occupational Health Services (OHS). Other Measures: The unit was terminally cleaned and daily enhanced cleaning with bleach ensued. Daily assessment of patients and staff for symptoms. Admissions were held for 2 days until all the patients were tested and given vaccine. Further admissions were screened for HAV.

**Results:**

32 inpatients screened. One patient was positive for HAV IgM, but was asymptomatic with normal LFTs. On investigation, patient had acute hepatitis in February 2021. Patients with no immunity were vaccinated. Two immunocompromised patients were also given HAV immunoglobulin. On chart review, 6 out of 29 discharged patients had evidence of immunity. 133 staff were screened and 54 referred to OHS (see table).

Exposure Investigation

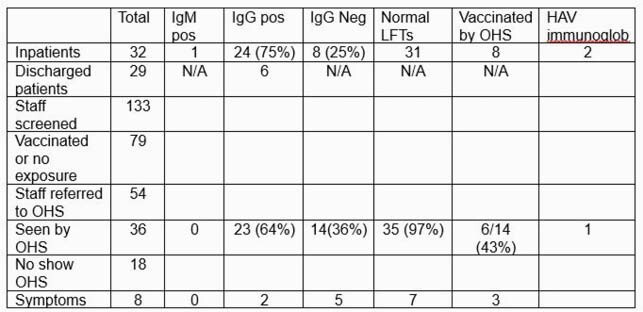

**Conclusion:**

As evident with numerous COVID outbreaks in inpatient Psychiatry units, communicable diseases are difficult to control. Patients are in an interactive communal setting and participate in group sessions. For better care and safety of patients and staff, our unit will screen and offer HAV vaccine to new admissions.

**Disclosures:**

**Gregory Weston, MD MSCR**, **Allergan** (Grant/Research Support) **Inessa Gendlina**, Nothing to disclose

